# Equivalent inductance model for the design analysis of electrodynamic suspension coils for hyperloop

**DOI:** 10.1038/s41598-021-02907-7

**Published:** 2021-12-06

**Authors:** Jungyoul Lim, Chang-Young Lee, Ye Jun Oh, Jeong-Min Jo, Jin-Ho Lee, Kwan-Sup Lee, Suyong Choi

**Affiliations:** New Transportation Innovative Research Center, Korea Railroad Research Institute, Uiwang-si, 16105 Gyeonggi-do Korea

**Keywords:** Electrical and electronic engineering, Mechanical engineering

## Abstract

Hyperloop is a new concept of ground transportation. In Hyperloop, travelling occurs in near-vacuum tubes under 0.001 atm at a subsonic speed of up to 1200 km/h. During acceleration to and driving at a subsonic speed, magnetic levitation is employed. Thus far, various levitation technologies in existing high-speed maglev trains have been considered. Among those technologies, superconducting (SC) electrodynamic suspension (EDS) is a highly effective levitation system for Hyperloop owing to its advantages of a large levitation gap, levitation stability, and control being unnecessary. However, analyzing an EDS system requires the electromagnetic transient analysis of complex three-dimensional (3D) features, and its computational load generally limits the use of numerical methods, such as the 3D finite element method (FEM) or dynamic circuit theory. In this study, a novel model that can rapidly and accurately calculate the frequency-dependent equivalent inductance was developed. The developed model was then applied to design an EDS system using the decoupled resistance-inductance equations of levitation coils. Next, levitation coils of SC-EDS were designed and analyzed for use in Hyperloop. The obtained results were compared with the FEM results to validate the developed model. In addition, the model was experimentally validated by measuring currents induced by moving pods.

## Introduction

Recently, magnetically levitated ultra-high-speed ground transportation, such as Hyperloop, has attracted significant attention worldwide^1–6^. Instead of a traditional wheel-rail system that has a speed limit, non-contact magnetic levitation technologies are being used in high-speed transportation systems. Magnetic levitation technologies for high-speed transport include electromagnetic suspension (EMS) and electrodynamic suspension (EDS). In EMS, a controlled attractive force between onboard electromagnets and ferromagnetic rails is utilized, and in EDS, an induced repulsive force between onboard magnets and conductive rails is utilized. The former has been implemented in official lines in Shanghai, China, since 2004^7^, and the latter will be implemented in commercial lines in Japan in 2027^8^. Furthermore, research has been conducted on self-stable magnetic levitation using the flux-pinning effect of high-temperature superconductors (SCs)^9,10^.

In Hyperloop, travelling occurs at velocities of up to 1200 km/h in a vacuum tube with low air resistance. To achieve such speeds, active levitation EMS that controls the force transmitted to steel vacuum tubes or rails can be implemented^11–13^. Alternatively, one can implement passive levitation EDS that uses the induced repulsive force acting on onboard magnets moving on conductive rails^14,15^. Hyperloop One^16^, which has recently succeeded in on-board passenger experiments at velocities of 173 km/h, switched from EDS levitation to EMS levitation. Each magnetic levitation method has its own advantages and disadvantages. Hyperloop, which shares superconducting electromagnets (SCMs) in the vehicle by EDS levitation and linear synchronous motor (LSM) propulsion, has the following advantages^17–20^. The strong magnetic field generated by SCMs generates a strong propulsion force to reach subsonic speeds, enables stable levitation at high-speed driving without control, and increases the levitation air gap, which can lower the infrastructure construction costs. EDS levitation with SCMs uses an efficient null-flux system with a high lift-to-drag ratio^21–24^. Such null-flux coils are typically arranged on the sidewalls of the guideway for vertical levitation and horizontal guidance of vehicles with SCMs by currents induced in the coils.

The design and analysis of the EDS system requires the electromagnetic transient analysis of complex three-dimensional (3D) features, which can be researched using general numerical methods such as 3D finite element method (FEM)^25–27^. These general numerical methods can achieve accurate results. However, the transient analysis of a moving vehicle along a linear inductive coil track incurs a high computational load and is generally limited to the analysis of a particular design or the validation of other analysis models. Therefore, the dynamic circuit theory^28,29^ is commonly used as a more efficient analysis method. In the dynamic circuit theory, electromagnetic elements located in space are modeled as time-dependent circuit parameters, and then, the system equations are solved. The electromagnetic interaction between SCMs and null-flux coils can be modeled as space- and time-dependent inductances. Thus, the EDS system can be directly analyzed by solving the ordinary differential equations (ODEs) of resistance–inductance (RL) circuits. However, computational loads arise from mutual inductance, which is a representation of the magnetic coupling between levitation coils and moving SCMs. For a rapid analysis using simplified system models, as achieved in previous studies, only the mutual inductance between electrically connected coils is considered, while the magnetic coupling effect due to adjacent coils is neglected^30–32^. In certain cases, only the fundamental waves of SCMs are considered for analysis. However, by ignoring the mutual inductance due to adjacent coils, analysis errors may occur, which can be added or subtracted depending on the coil geometry. For polyphase rotary electrical machines, an inductance matrix, which can cause difficulties in analysis and control, is transformed to a magnetically decoupled system based on eigenvalues^33,34^. However, no research has been conducted on the linear arrangement of multiple null-flux coils along an EDS track. Lim et al. recently proposed a rapid design model^35^ that enables efficient analyses of the decoupled RL equations of levitation coils by determining a constant effective inductance that includes all the coupling effects of adjacent coils. However, accurate analysis data or experimental results are required to extract the effective inductance of the system.

To address the aforementioned problems, this paper presents an equivalent inductance model (EIM) that determines the equivalent inductance from RL equations for each isolated coil of a magnetically decoupled EDS system. The EIM utilizes the characteristic of induced current and electromotive force (EMF) in levitation coils arranged at regular intervals along the guideway. The decoupled RL equations by the EIM can be solved rapidly and accurately using the Fourier series for the EMF. After describing the EIM of normal- and null-flux coils in the EDS system, levitation coils of superconducting (SC) EDS were designed and analyzed for use in Hyperloop. In addition, to validate the EIM, the analyzed results for various designs were compared with those of the finite element model (FEM). Furthermore, the induced currents for two types of moving pods, namely, SCMs and permanent magnets (PMs), on a small-scale testbed were compared with the analysis results.

## Methods

### Inductance model for normal-flux coils

**Figure 1:**
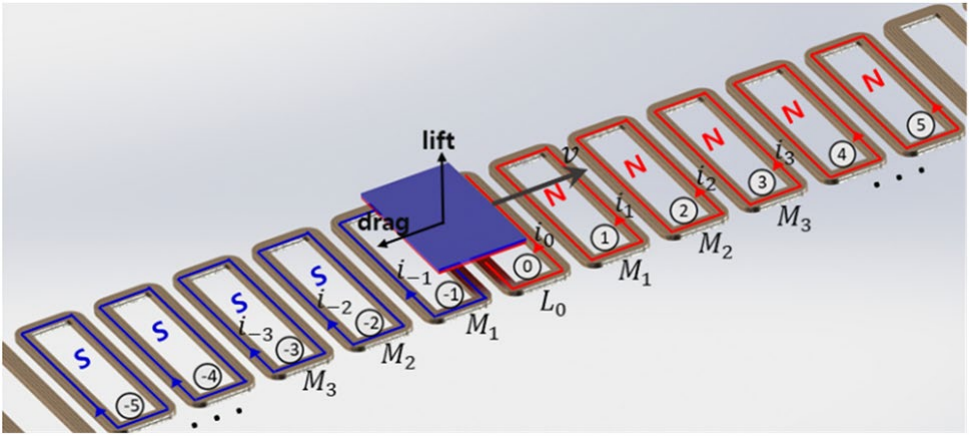
Inductances and induced currents on a discrete EDS coil track and lift/drag forces acting on a moving magnet.

An EDS system uses a repulsive force applied to a magnet on a vehicle by a conductive track. As shown in Fig. 1, when considering an EDS system in which a magnet is moving at velocity ${\varvec{v}}$ on levitation coils indexed by $p=\cdots , -1, 0, 1, \cdots$, currents are induced in the coils to interfere with the movement of the magnet. The coils magnetize into the north (N) or south (S) poles by the induced currents that produce lift and drag forces acting on the magnet.

If an arbitrary coil is selected as the *0*-th coil denoted by $p=0$ in the coil track, other coils are considered to be located at pitch ${\tau }_{c}$ intervals in both directions from the *0*-th coil. When the *p*-th coil represents a coil located on ${p\tau }_{c}$ from the *0*-th coil, the pairs of the *0*-th and ± *p*-th coils have the same mutual inductance ${M}_{p}$. When the magnet is fixed at the origin and the coils move at $-{v}_{x}$ along the x-direction, the RL equation for the *0*-th coil located at $x=-{v}_{x}t$ can be expressed as follows:

1 $$- v_{x} L_{0} \frac{d}{dx}i_{0} - v_{x} \mathop \sum \limits_{p = 1}^{\infty } \left( {M_{p} \frac{d}{dx}\left( {i_{p} + i_{ - p} } \right)} \right) + Ri_{0} = \varepsilon_{0}$$

where ${i}_{0}$ and ${i}_{p}$ denote the induced current at the *0*-th and *p*-th coils, respectively, and ${\varepsilon }_{0}$ denotes the EMF at the *0*-th coil. For the identical resistance $R$ and self-inductance ${L}_{0}$ for each coil, the induced EMF ${\varepsilon }_{p}$ and current ${i}_{p}$ of the *p*-th coil, which are caused by the relative motion between the magnet and coil, become the shift of the ${\varepsilon }_{0}$ and ${i}_{0}$ functions for the *0*-th coil position $x$ by $-p{\tau }_{c}$:

2a $${\varepsilon }_{p}\left(x\right)={\varepsilon }_{0}\left(x+p{\tau }_{c}\right)$$

2b $${i}_{p}\left(x\right)={i}_{0}\left(x+p{\tau }_{c}\right)$$

When an even number of onboard magnets are paired with the N and S poles, the induced EMF of the *0*-th coil ${\varepsilon }_{0}$ is an even function. For coil position $x$ in the ${2\tau }_{0}$ interval, the Fourier frequency ${\omega }_{n}$ is defined as follows:

3 $${\omega }_{n}=-\pi n/{\tau }_{0}$$

With the Fourier coefficient ${A}_{n}$, ${\varepsilon }_{0}$ can be represented by the Fourier cosine series in Eq. (4). In the case of an odd number of onboard magnets, the odd function ${\varepsilon }_{0}$ can be expressed as a Fourier sine series:

4 $$\varepsilon_{0} \left( x \right) = \mathop \sum \limits_{n = 1}^{\infty } \varepsilon_{0n} \;\;\;\;where\;\;\varepsilon_{0n} = v_{x}A_{n} cos\left( {\omega_{n} x} \right)$$

In addition, the induced current at the *p*-th coil can be expressed in the general form of the Fourier series for coefficients ${B}_{n}$ and ${C}_{n}$:

5 $$i_{p} \left( x \right) = \mathop \sum \limits_{n = 1}^{\infty } i_{pn} ,\;\;\;where\;\;\;i_{pn} = B_{n} \cos \left( {\omega_{n} \left( {x + p\tau_{c} } \right)} \right) + C_{n} sin\left( {\omega_{n} \left( {x + p\tau_{c} } \right)} \right)$$

Using Eq. (5) and the trigonometric sum-to-product identities, the sum of the induced currents of the $\pm p$-th coil pair can be explicitly expressed as a function of the induced current on the *0*-th coil ${i}_{0n}={B}_{n}\mathit{cos}\left({\omega }_{n}x\right)+{C}_{n}sin({\omega }_{n}x)$:

6 $$i_{pn} + i_{ - pn} = 2\cos \left( {\omega_{n} p\tau_{c} } \right)i_{0n}$$

By applying Eq. (6) to Eq. (1), the RL equation of the *n*-th harmonic for the *0*-th coil can be obtained as follows:

7 $$- v_{x} \left( {L_{0} + 2\mathop \sum \limits_{p = 1}^{\infty } M_{p} \cos \left( {\omega_{n} p\tau_{c} } \right)} \right)\frac{d}{dx}i_{0n} + Ri_{0n} = \varepsilon_{0n} ,$$

where the *0*-th coil is magnetically decoupled with other coils. Therefore, considering that mutual inductance decreases rapidly with distance, the magnetic effects of adjacent coils at a specific *n*-th harmonic can be replaced by the equivalent inductance ${L}_{e}$ in Eq. (8), or explicitly represented by the ratio of interval ${2\tau }_{0}$ and coil pitch ${\tau }_{c}$ using Eq. (3).

8 $${L}_{e}(n)={L}_{0}+{\sum }_{p=1}^{{P}_{e}}2{M}_{p}\mathit{cos}\left({\omega }_{n}p{\tau }_{c}\right)={L}_{0}+{\sum }_{p=1}^{{P}_{e}}2{M}_{p}\mathit{cos}\left(pn\pi \frac{{\tau }_{c}}{{\tau }_{0}}\right)$$

In this equation, ${P}_{e}$ is the number of pairs of adjacent coils to be included in ${L}_{e}$ and ${L}_{e}=$ ${L}_{0}$ for ${P}_{e}=0$. Moreover, ${L}_{0}$ and ${M}_{p}$ can be obtained either experimentally or using static numerical analysis methods. With the decoupled RL Eq. (7), the induced current of the *p*-th coil ${i}_{pn}$ expressed in closed-form solutions^35^ can be calculated directly as follows:

9a, 9b $${B}_{n}=\frac{{A}_{n}v_{x}\mathrm{cos}({\mathrm{\varphi }}_{\mathrm{n}})}{\sqrt{{R}^{2}+{\left({v}_{x}{\omega }_{n}{L}_{e}\right)}^{2}}}, {C}_{n}=-\frac{{A}_{n}v_{x}\mathrm{sin}({\mathrm{\varphi }}_{\mathrm{n}})}{\sqrt{{R}^{2}+{\left({v}_{x}{\omega }_{n}{L}_{e}\right)}^{2}}}$$

9c $${i}_{pn}=\frac{{A}_{n}{v}_{x}}{\sqrt{{R}^{2}+{\left({v}_{x}{\omega }_{n}{L}_{e}\right)}^{2}}}\mathit{cos}\left({\omega }_{n}x+{\varphi }_{p}+{\varphi }_{n}\right)$$

where ${\varphi }_{p}={\omega }_{n}p{\tau }_{c}$ and ${\mathrm{\varphi }}_{\mathrm{n}} \, ={\mathrm{tan}}^{-1}\frac{{{v_{x}}\upomega }_{\mathrm{n}}{\mathrm{L}}_{e}}{\mathrm{R}}$ represent the phase shifts due to the *p*-th coil position and coil inductance, respectively.

### Inductance model for null-flux coils

**Figure 2:**
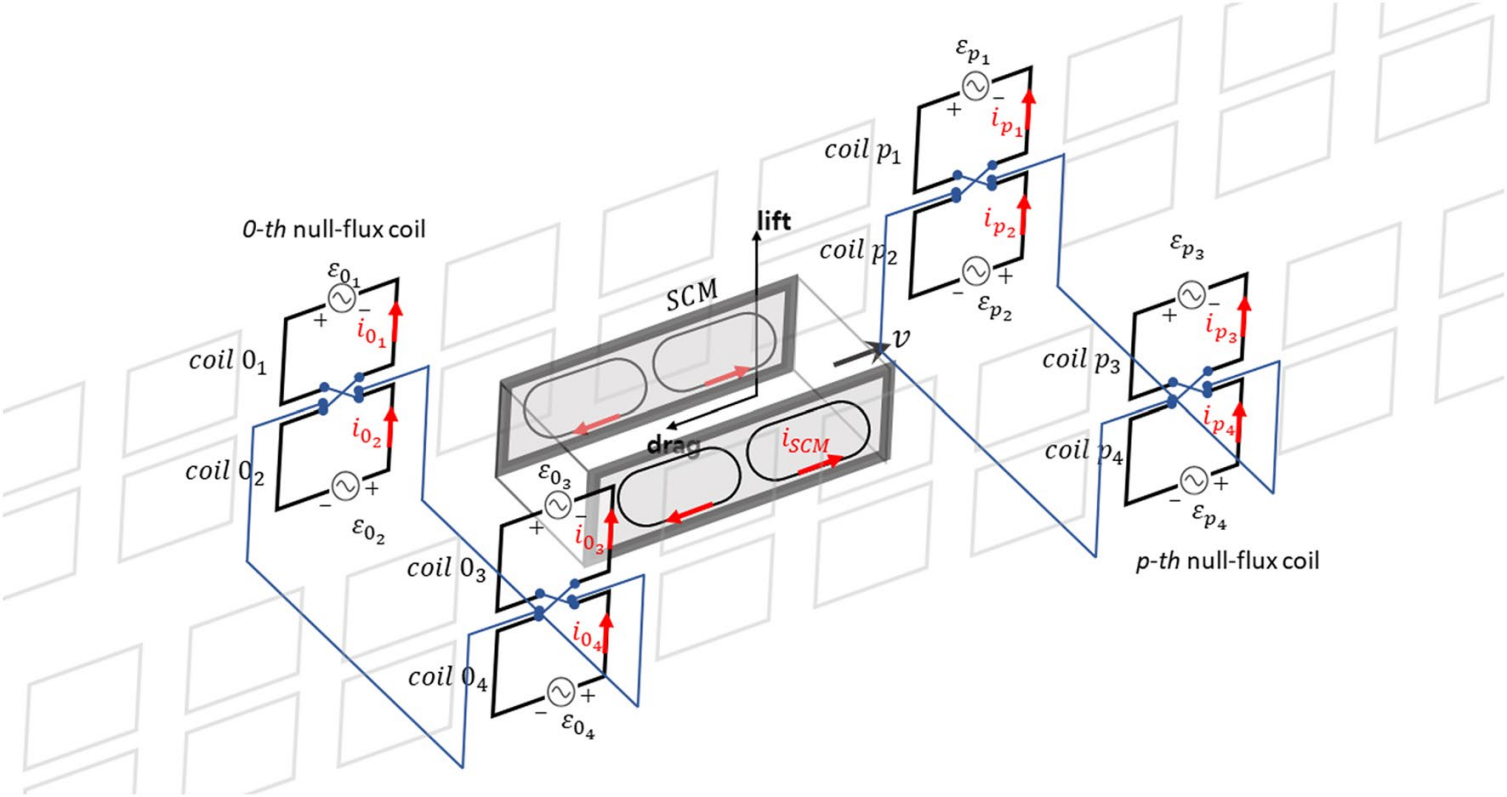
Schematic of an SCM pod moving on a null-flux EDS track and configuration of a null-flux coil consisting of four electrically connected coils.

Using the EIM for normal-flux coils, the equivalent inductance on a null-flux EDS track can be readily determined. Figure 2 presents null-flux coils attached to a sidewall and a moving SCM pod, which has two SCMs consisting of two poles with a current ${i}_{SCM}$. A set of null-flux coils composed of four coils placed on both sidewalls are electrically connected such that all the induced EMFs are canceled at the center. Thus, the induced EMFs and restoring forces are proportional to the vertical and horizontal displacements from the center. As in the case of normal-flux coils, the *0*-th coil can be arbitrarily selected, and the *p*-th coil located relatively on $p{\tau }_{c}$ from the *0*-th coil can be considered. The four coils of the *p*-th null-flux coil are numbered by subscripts *k* = 1–4 from the top left to the bottom right, and ${\varepsilon }_{{p}_{k}}$ and ${i}_{{p}_{k}}$ denote the induced EMF and current of the ${p}_{k}$-th coil, respectively.

The coils on the same sidewall are located close to each other and magnetically influence each other, whereas the magnetic interaction of the coils located on both sidewalls can be ignored. Considering coils 0 and *p* on the same sidewall, the mutual inductance between the coils at the same and different vertical positions can be respectively represented by ${M}_{pa}$ and ${M}_{pb}$. When the self-inductance of a coil is ${L}_{s}$ and the mutual inductance between electrically connected vertical coils is ${M}_{0}$, the RL equation for the *0*-th null-flux coil has a similar form as Eq. (1) for mutual and self-inductance matrices $\mathbf{M}$ and $\mathbf{L}$ as follows:

10 $$- v_{x} {\mathbf{L}}\frac{d}{dx}{\varvec{i}}_{0} - v_{x} \mathop \sum \limits_{p = 1}^{\infty } {\mathbf{M}}\frac{d}{dx}\left( {{\varvec{i}}_{p} + {\varvec{i}}_{ - p} } \right) + R{\varvec{i}}_{0} = {\varvec{\varepsilon}}_{0} ,$$

where the induced current vector $${{\varvec{i}}}_{p}={\left[\begin{array}{cc}\begin{array}{cc}{i}_{{p}_{1}}& {i}_{{p}_{2}}\end{array}& \begin{array}{cc}{i}_{{p}_{3}}& {i}_{{p}_{4}}\end{array}\end{array}\right]}^{T}$$; the induced EMF vector $${{\varvec{\varepsilon}}}_{0}={\left[\begin{array}{cc}\begin{array}{cc}{\varepsilon }_{{0}_{1}}& {\varepsilon }_{{0}_{2}}\end{array}& \begin{array}{cc}{\varepsilon }_{{0}_{3}}& {\varepsilon }_{{0}_{4}}\end{array}\end{array}\right]}^{T}$$;$$\mathbf{M}=\left[\begin{array}{cc}{{\varvec{M}}}_{{\varvec{p}}}& {{\varvec{O}}}_{2}\\ {{\varvec{O}}}_{2}& {{\varvec{M}}}_{{\varvec{p}}}\end{array}\right];\mathrm{and}{{\varvec{M}}}_{{\varvec{p}}}=\left[\begin{array}{cc}{M}_{pa}& {M}_{pb}\\ {M}_{pb}& {M}_{pa}\end{array}\right]$$. Moreover, $$\mathbf{L}=\left[\begin{array}{cc}{{\varvec{L}}}_{0}& {{\varvec{O}}}_{2}\\ {{\varvec{O}}}_{2}& {{\varvec{L}}}_{0}\end{array}\right],\mathrm{where}{{\varvec{L}}}_{0}=\left[\begin{array}{cc}{L}_{s}& 0\\ 0& {M}_{0}\end{array}\right]$$ and ${{\varvec{O}}}_{2}$ is a 2 × 2 zero matrix. For each sidewall on the *p*-th null-flux coil, ${{\varvec{i}}}_{p}$ can be expressed separately by the current ${{\varvec{i}}}_{pL}$, which is equal in size and opposite in direction, and ${{\varvec{i}}}_{pG}$, which is equal in size and direction:

11 $${{\varvec{i}}}_{p}={{\varvec{i}}}_{pL}+{{\varvec{i}}}_{pG},$$

where $${{\varvec{i}}}_{pL}={\left[\begin{array}{cc}\begin{array}{cc}{i}_{{p}_{a}L}& -{i}_{{p}_{a}L}\end{array}& \begin{array}{cc}{i}_{{p}_{b}L}& -{i}_{{p}_{b}L}\end{array}\end{array}\right]}^{T};$$ $${{\varvec{i}}}_{pG}={\left[\begin{array}{cc}\begin{array}{cc}{i}_{{p}_{a}G}& {i}_{{p}_{a}G}\end{array}& \begin{array}{cc}{i}_{{p}_{b}G}& {i}_{{p}_{b}G}\end{array}\end{array}\right]}^{T}$$; ${i}_{{p}_{a}L}=({i}_{{p}_{1}}-{i}_{{p}_{2}})/2$; ${i}_{{p}_{b}L}=({i}_{{p}_{3}}-{i}_{{p}_{3}})/2$; ${i}_{{p}_{a}G}=({i}_{{p}_{1}}+{i}_{{p}_{2}})/2$; and ${i}_{{p}_{b}G}=({i}_{{p}_{3}}+{i}_{{p}_{3}})/2$. In addition, applying Kirchhoff’s current law to any node yields the following:

12 $${i}_{{p}_{a}G}=-{i}_{{p}_{b}G}$$

The equation for ${{\varvec{i}}}_{pL}$ is obtained by applying Eqs. (11) to (10) and by subsequently removing ${{\varvec{i}}}_{pG}$ using subtracting rows. The result is as follows:

13 $$- v_{x} \left( {L_{s} - M_{0} } \right)\frac{d}{dx}{\varvec{i}}_{0L} - v_{x} \mathop \sum \limits_{p = 1}^{\infty } \left( {M_{pa} - M_{pb} } \right)\frac{d}{dx}\left( {{\varvec{i}}_{pL} + {\varvec{i}}_{ - pL} } \right) + R{\varvec{i}}_{0L} = {\varvec{\varepsilon}}_{0L} ,$$

where ${{\varvec{\varepsilon}}}_{0L}={\mathbf{A}}_{L}{{\varvec{\varepsilon}}}_{0}$ denotes the induced EMF for ${{\varvec{i}}}_{pL}$ and $${\mathbf{A}}_{L}=\frac{1}{2}\left[\begin{array}{ccc} 1& -1& \begin{array}{cc} 0& 0\end{array}\\ -1& 1& \begin{array}{cc} 0& 0\end{array}\\ \begin{array}{c} 0\\ 0\end{array}& \begin{array}{c} 0\\ 0\end{array}& \begin{array}{cc}\begin{array}{c} 1\\ -1\end{array}& \begin{array}{c}-1\\ 1\end{array}\end{array}\end{array}\right]$$.

From ${{\varvec{\varepsilon}}}_{0L}$, ${{\varvec{i}}}_{pL}$ induced by the EMF difference between the cross-connected vertical coils generates a levitation force to the SCM pod. Comparing Eqs. (13) and (1), ${L}_{0}={L}_{s}-{M}_{0}$ and ${M}_{p}={M}_{pa}-{M}_{pb}$, and the equivalent inductance ${L}_{eL}$ is expressed as follows:

14 $${L}_{eL}(n)=({L}_{s}-{M}_{0})+{\sum }_{p=1}^{P_e}2({M}_{pa}-{M}_{pb})\mathit{cos}\left(pn\pi \frac{{\tau }_{c}}{{\tau }_{0}}\right)$$

In addition,${{\varvec{i}}}_{pG}$ is obtained by applying Eqs. (11) and (12) to Eq. (10) and then removing ${{\varvec{i}}}_{pL}$ by adding rows. The result is as follows:

15 $$-{v}_{x}\left({L}_{s}+{M}_{0}\right)\frac{d}{dx}{{\varvec{i}}}_{0G}-{v}_{x}{\sum }_{p=1}^{\infty }\left({M}_{pa}+{M}_{pb}\right)\frac{d}{dx}\left({{\varvec{i}}}_{pG}+{{\varvec{i}}}_{-pG}\right)+R{{\varvec{i}}}_{0G}={{\varvec{\varepsilon}}}_{0G}$$

where ${{\varvec{\varepsilon}}}_{0G}={\mathbf{A}}_{G}{{\varvec{\varepsilon}}}_{0}$ denotes the induced EMF for ${{\varvec{i}}}_{pG}\mathrm{ and}$ $${\mathbf{A}}_{G}=\frac{1}{4}\left[\begin{array}{ccc} 1& 1& \begin{array}{cc}-1& -1\end{array}\\ 1& 1& \begin{array}{cc}-1& -1\end{array}\\ \begin{array}{c}-1\\ -1\end{array}& \begin{array}{c}-1\\ -1\end{array}& \begin{array}{cc} \begin{array}{c} 1\\ 1\end{array}& \begin{array}{c} 1\\ 1\end{array}\end{array}\end{array}\right]$$.

Similarly, from ${{\varvec{\varepsilon}}}_{0G}$, ${{\varvec{i}}}_{pG}$ induced by the EMF difference between the null-flux connected horizontal coils generates a guidance force to the SCM pod. Comparing Eqs. (15) and (1), ${L}_{0}={L}_{s}+{M}_{0}$, ${M}_{p}={M}_{pa}+{M}_{pb}$, and the equivalent inductance ${L}_{eG}$ is expressed as follows:

16 $${L}_{eG}(n)=({L}_{s}+{M}_{0})+\sum _{p=1}^{P_e}2({M}_{pa}+{M}_{pb})\mathit{cos}\left(pn\pi \frac{{\tau }_{c}}{{\tau }_{0}}\right)$$

Therefore, ${{\varvec{i}}}_{pL}$ and ${{\varvec{i}}}_{pG}$ can be easily determined by applying ${{\varvec{\varepsilon}}}_{0L}$, ${{\varvec{\varepsilon}}}_{0G},$ ${L}_{eL}$, and ${L}_{eG}$ to Eqs. (9a–9c), and the induced current ${{\varvec{i}}}_{p}$ can be obtained by summing the two currents, as expressed by Eq. (11).

### Induced EMF and force equation for EDS coils

When the magnets move at ${\varvec{v}}={\left[{v}_{x} {v}_{y} {v}_{z}\right]}^{T}$, the coils can be considered to move at $-{\varvec{v}}$ instead of the magnets. In this case, the induced EMF $\varepsilon$ and force ${{\varvec{f}}}_{(\mathrm{lev})}$ on a coil can be determined by the induced current *I* and EMF vector ${\varvec{b}}$^29,35^:

17 $$\varepsilon ={{\varvec{b}}}^{T}\boldsymbol{ }{\varvec{v}}$$

18 $${{\varvec{f}}}_{(lev)}={\varvec{b}}I$$

where ${\varvec{b}}={\left[{b}_{(x)} {b}_{(y)} {b}_{(z)}\right]}^{T}$ is defined by Faraday’s law for moving conductors. For the unit directional vector ${{\varvec{e}}}_{(d)} (d=x,y,z)$, a line integral following wire loop C on the coil yields ${b}_{(d)}$ as follows:

19 $${b}_{(d)}=-{\oint }_{C}{{\varvec{e}}}_{(d)}\times {\varvec{B}}\left({\varvec{r}}\right)\bullet d{\varvec{l}}$$

where ${\varvec{B}}\left({\varvec{r}}\right)$ is the flux density of the magnet at the wire position $\mathbf{r}$ and $\mathrm{d}{\varvec{l}}$ is an infinitesimal vector element of wire loop C.

Based on the force acting on the moving coil, as expressed by Eq. (18), the total force acting on the moving magnet can be calculated by the sum of the forces acting on the *k*-th coil of the *p*-th null-flux coil when the EDS track consists of $2{P}_{0}+1$ null-flux coils:

20 $${\varvec{f}}=-\sum_{p=-{P}_{0}}^{{P}_{0}}\sum_{k=1}^{4}{{\varvec{f}}}_{{p}_{k}(lev)}$$

For a more rapid calculation of ${\varvec{b}}$, virtual ${n}_{turn}={n}_{w}\times {n}_{t}$ turns of closed loop ${C}_{i}$ are considered instead of the actual wire loops with ${N}_{turn}={N}_{w}\times {N}_{t}$ turns. Thereafter, ${b}_{(d)}$ can be approximated by the sum of ${b}_{i(d)}$ calculated for each ${C}_{i}$ as follows:

21 $${b}_{(d)}\approx ({N}_{turn}/{n}_{turn})\sum_{i=1}^{{n}_{turn}}{b}_{i(d)}$$

In addition, when ${C}_{i}$ is divided into ${n}_{ij}$ small line segments, the induced EMF vector ${b}_{i}$ of ${C}_{i}$ can be approximated by the Riemann sum as follows:

22 $${b}_{i(d)}\approx \sum_{j=1}^{{n}_{ij}}{{\varvec{e}}}_{(d)}\times {\varvec{B}}\left({{\varvec{r}}}_{ij}\right)\bullet {{\varvec{l}}}_{ij}$$

where ${{\varvec{r}}}_{{\varvec{i}}{\varvec{j}}}$ and ${{\varvec{l}}}_{ij}$ denote the center position and length of the $j$-th segment on ${C}_{i}$, respectively.

## Results

To demonstrate the effectiveness of the EIM, null-flux levitation coils for small-scale Hyperloop testbeds were designed. Herein, first, the design parameters for an SCM and null-flux coils are described. Next, the results of the performance analysis are presented for various designs.

Three designs were selected based on the performance criteria, and detailed performance metrics such as the magnetic force and stiffness were compared under various operating conditions, including varying pod mass and velocity. Furthermore, to validate the accuracy of the model, the results obtained using the EIM were compared with the results obtained using the FEM. The magnetic field of the magnet, inductance of the coil, and analysis results for validation were computed using Simcenter MagNet 2021.1^36^, which is a commercial FEM software.

### Design parameters of EDS coils for hyperloop

**Figure 3:**
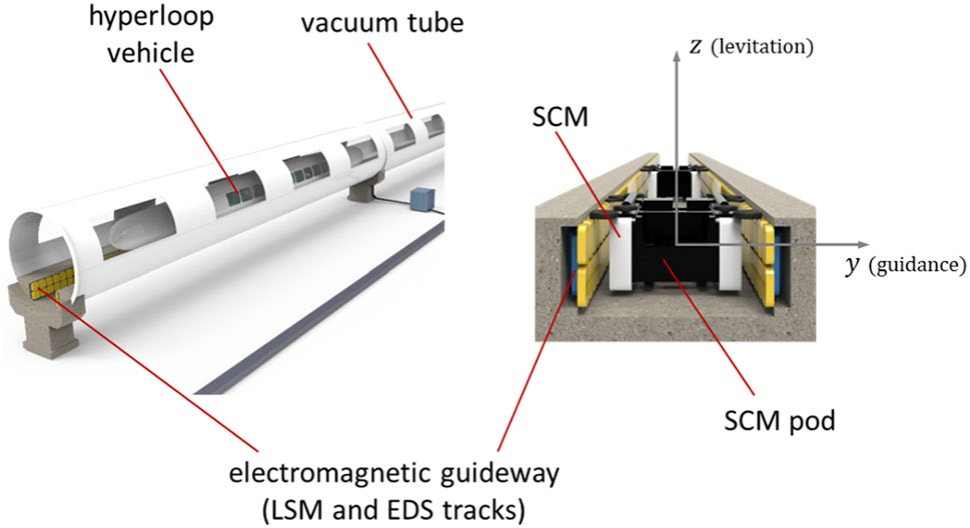
Concept of the Hyperloop vehicle with SCM pods in a vacuum tube with an electromagnetic guideway consisting of LSM and EDS tracks.

Figure 3 illustrates the concept of Hyperloop, in which a hyperloop vehicle travels at subsonic speeds along an electromagnetic guideway in a vacuum tube. A pair of SCMs is mounted on both sides of the pod supporting the Hyperloop vehicle, and the SCM pod is driven in the x-direction by the LSM installed on the guideway. In addition, the pod is levitated and guided by null-flux EDS on the guideway, which generates restoring lift and guidance forces as functions of the vertical z- and horizontal y-directional displacements. For a small-scale testbed of Hyperloop, we designed null-flux coils to illustrate the effectiveness of the EIM.

**Figure 4:**
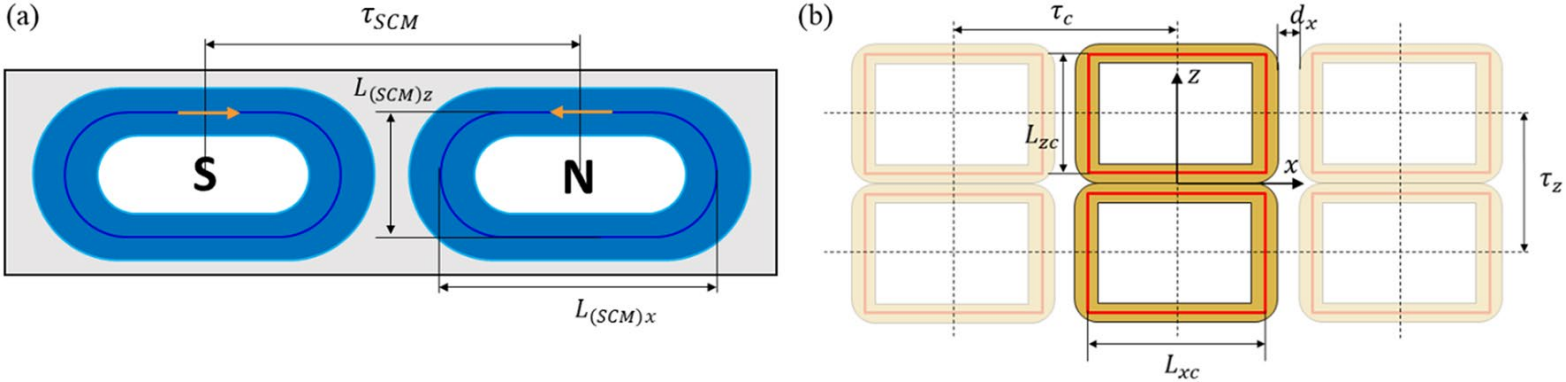
Design layouts of the (**a**) SCM module and (**b**) null-flux coil for design examples and model validation.

Figure 4 presents the design layout of a small-scale SCM module and a null-flux levitation coil for industrial applications. For the testbed, each module of the SCM, consisting of two poles separated by pole pitch ${\tau }_{SCM}$, was set to the center size of ${L}_{\left(SCM\right)x}\times {L}_{\left(SCM\right)z}$ and magnetomotive force (MMF). Each null-flux coil was separated by pitch ${\tau }_{c}$. The center size of the coil was ${L}_{xc}\times {L}_{zc}$, and the coil gap along the moving direction was ${d}_{x}$. In addition, the multiturn null-flux coil had a number of turns ${N}_{turn}$, which consisted of ${N}_{w}$ turns in the xz plane and ${N}_{t}$ turns along the y-axis with wire thicknesses ${c}_{w}$ and ${c}_{t}$, respectively. The detailed design parameters and variables are summarized in Table 1. Given that the physical air gap was ${g}_{air}$ and the thickness of the levitation coil was ${L}_{t}$, the performance of the levitation coil design was evaluated at a subsonic driving velocity ${v}_{h}$ and/or a take-off velocity ${v}_{l}$. Based on these design layouts and parameters, the performance with respect to various ${N}_{turn}$ and ${L}_{zc}$ values was analyzed for three horizontal pitches. Subsequently, the design with the preferred performance for each pitch was selected and analyzed.

**Table 1:** Parameters of SCM and null-flux levitation coils for design examples and model validation.

Parameter		Value	Unit
SCM	Pole pitch, ${\tau }_{SCM}$	$0.81$	$\mathrm{m}$
	Coil size, ${L}_{\left(SCM\right)x}\times {L}_{\left(SCM\right)z}$	$0.6\times 0.3$	${\mathrm{m}}^{2}$
	Magnetomotive force	300	$\mathrm{kAt}$
Levitation coil	Number of turns, ${N}_{turn}={N}_{w}\times {N}_{t}$	${N}_{t}=2, {N}_{w}=2,\dots , 15$	-
	Horizontal pitch, ${\tau }_{c}$	$(1/3, 1/2, 2/3)\times {\tau }_{SCM}$	$\mathrm{m}$
	Vertical coil height, ${L}_{zc}$	$0.2, 0.21, \dots , 0.39, 0.4$	$\mathrm{m}$
	Wire cross sectional area, ${A}_{w}={c}_{w}\times {c}_{t}$	$0.01\times 0.01$	${\mathrm{m}}^{2}$
	Horizontal gap between coils, ${d}_{x}$	$0.03$	$\mathrm{m}$
	Horizontal coil width, ${L}_{xc}$	${\tau }_{c}-{d}_{x}-{c}_{w}{N}_{w}$	$\mathrm{m}$
	Vertical pitch, ${\tau }_{z}$	${L}_{zc}+{c}_{w}{N}_{w}$	$\mathrm{m}$
	Aluminum wire resistivity, ${\rho }_{alu}$	$2.8265\times {10}^{-8}$	$\Omega \bullet \mathrm{m}$
	Air gap, ${g}_{air}$	$0.05$	$\mathrm{m}$
	Thickness of the levitation coil, ${L}_{t}$	$0.03$	$\mathrm{m}$
	Subsonic driving velocity, ${v}_{h}$	$277.78 (1000)$	m/s ($\mathrm{km}/\mathrm{h}$)
	Take-off velocity, ${v}_{l}$	$41.67 (150)$	m/s ($\mathrm{km}/\mathrm{h})$
	Maximum vertical displacement, ${\Delta z}_{max}$	$0.10$	$\mathrm{m}$
	Maximum horizontal displacement, ${\Delta y}_{max}$	$0.05$	$\mathrm{m}$

### Design results for null-flux EDS coils

**Figure 5:**
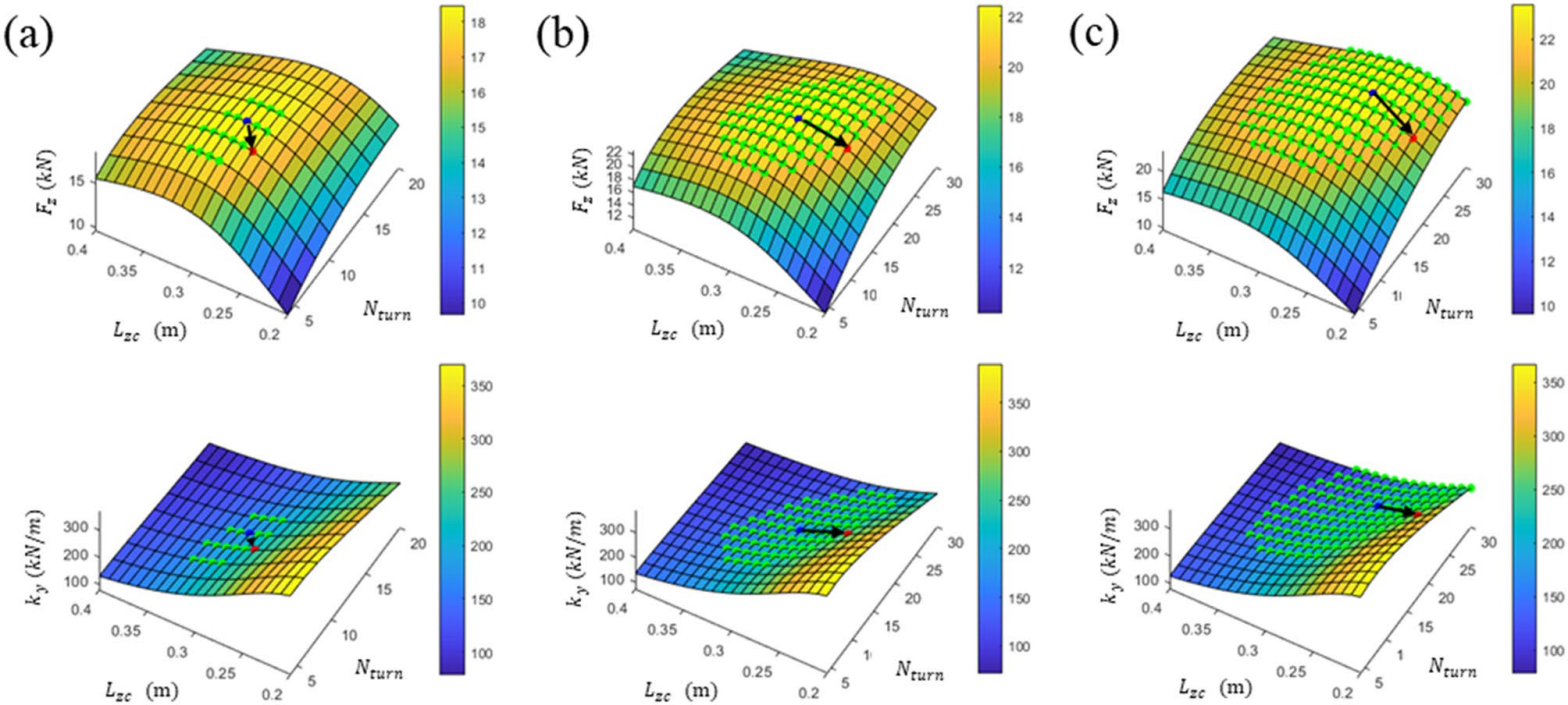
Design of null-flux coils with analyzed lift force at ${P}_{zm}$ and guidance stiffness at ${P}_{zh}$ with respect to changes in ${N}_{turn}$ and ${L}_{zc}$ at the selected pole pitches: (**a**) ${\tau }_{c}=(1/3){\tau }_{SCM}$, (**b**) ${\tau }_{c}=(1/2){\tau }_{SCM}$, and (**c**) ${\tau }_{c}=(2/3){\tau }_{SCM}$.

The analysis results of the lift force ${F}_{z}$ and horizontal stiffness ${k}_{y}$ of three types of levitation coil pitches with respect to changes in ${N}_{turn}$ and ${L}_{zc}$ at ${v}_{h}$ are shown in Fig. 5. To represent each pair of horizontal and vertical displacements as ($\Delta \mathrm{y}, \Delta \mathrm{z})$, ${F}_{z}$ and ${k}_{y}$ were evaluated at the maximum vertical displacement ${P}_{zm}=(0, -{\Delta z}_{max})$ and the intermediate vertical displacement ${P}_{zh}=(0, -{\Delta z}_{max}/2)$. A simple method for selecting a design is to maximize the lift force indicated by black dots on each graph. However, such selections can lead to an excessively low horizontal stiffness^37^. If a gentle characteristic of the ${F}_{z}$ surface near the peak point is utilized, it can compensate for the low horizontal stiffness by reducing ${F}_{z}$ to a certain level. Therefore, if the maximum ${F}_{z}$ of each pole pitch is ${F}_{zm}$ and the acceptable ${F}_{z}$ reduction ratio is ${\alpha }_{zm}$, a design that maximizes the horizontal stiffness with greater ${F}_{z}$ values than $(1-{\alpha }_{zm}){F}_{zm}$, as indicated by green dots, can be selected from each pitch. Considering that ${F}_{zm}$ is significantly different for each pitch ratio, ${\alpha }_{zm}$ was applied differently by 0.02, 0.06, and 0.10. The newly selected designs in Fig. 5 are indicated by a red dot, where ${L}_{zc}$ or the vertical pitch ${\tau }_{z}$ is reduced and ${N}_{turn}$ is reduced for several designs. Thus, when Designs A0, B0, and C0, indicated by black dots, were the original designs that maximized ${F}_{z}$ at each pole pitch, Designs A1, B1, and C1, indicated by red dots, were the corresponding improved designs that enhanced the horizontal stiffness from each original design. The shape parameters and performance of the designs are summarized in Table 2. For each pole pitch, Designs A1, B1, and C1 demonstrated considerably improved horizontal stiffnesses to 22.7%, 41.6%, and 56.4% at ${P}_{zh}$ instead of only 1.7%, 5.6%, and 9.0% ${F}_{z}$ reductions at ${P}_{zm}$, respectively. In addition, the levitation and guidance performances of B1 and C1 were similar.

**Table 2:** Comparison of the parameters and performances between designs that maximize the levitation force and designs with improved horizontal stiffness.

Design	${\tau }_{c}/{\tau }_{SCM}$	${N}_{turn}$	${L}_{zc}$ (m)	${\tau }_{z}$ (m)	${F}_{z}$ (kN) at ${P}_{zm}$	${k}_{y}$ (kN/m) at ${P}_{zh}$
A0	1/3	12	0.30	0.36	18.46	171.76
A1		10	0.28	0.33	18.15	210.81
B0	1/2	18	0.29	0.38	22.44	175.11
B1		18	0.24	0.33	21.18	247.91
C0	2/3	24	0.27	0.39	23.56	163.21
C1		22	0.22	0.33	21.44	255.32

**Figure 6:**
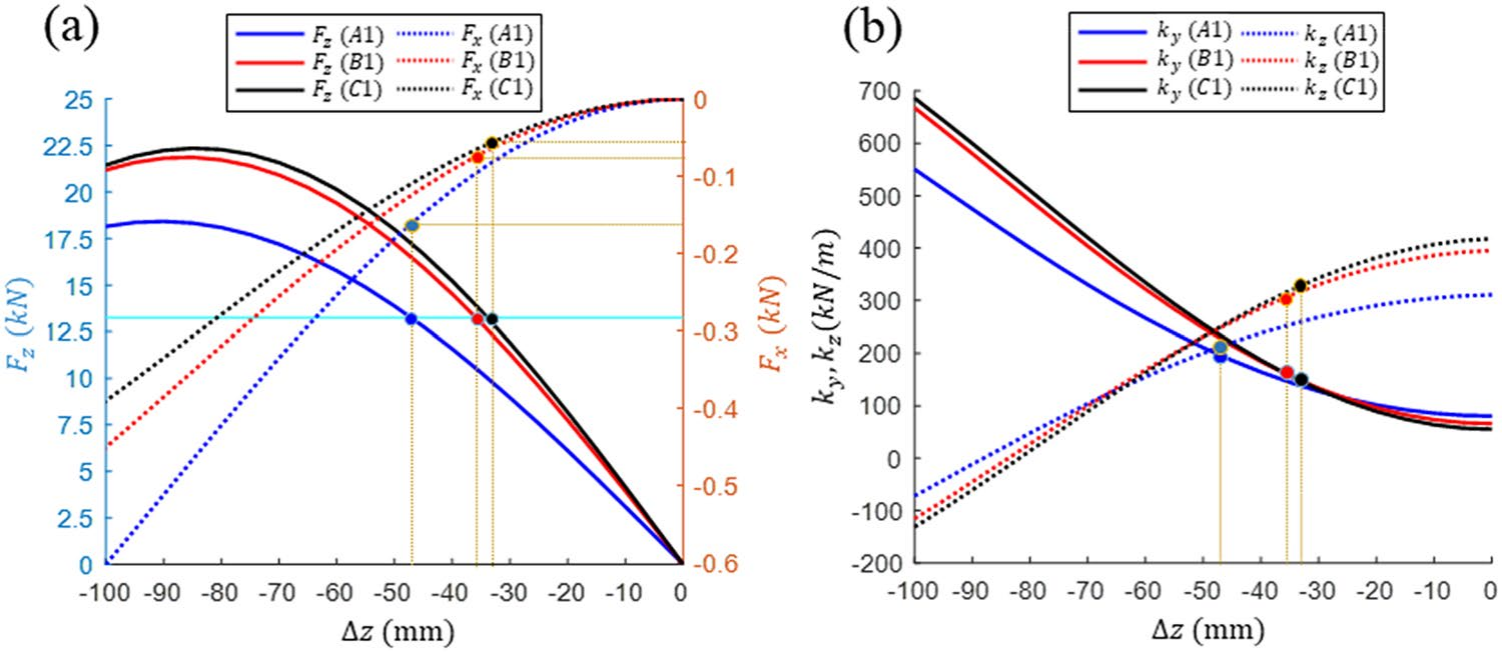
Comparison of the design characteristics with respect to the vertical displacement. (**a**) Lift and drag forces and (**b**) horizontal and vertical stiffnesses.

The performances and characteristics of the improved designs of A1, B1, and C1 were analyzed and compared in detail. First, when driving at velocity ${v}_{h}$, as shown in Fig. 6, the lift and drag forces and the horizontal and vertical stiffnesses according to the vertical displacement at the driving horizontal center ($\Delta \mathrm{y}=0$) were compared. Designs B1 and C1 demonstrated highly similar performances; however, the forces and stiffnesses of Design A1 were relatively lower at the same value of ∆z. As the vertical displacement was increased, the lift force gradually increased. However, the vertical stiffness defined by the slope of the lift force decreased gradually, thus resulting in maximum ∆z = –85 ~ –90 mm. There was a gradual increase in the drag force and the horizontal stiffness initially as $\Delta z$ was decreased, and then, a gradual increase to a considerably high value occurred. To analyze the driving characteristics of a design in terms of the lift-to-drag (LD) ratio, for a small vertical displacement, the LD ratio should be high, thus allowing for a higher driving efficiency based on magnetic levitation. For large vertical displacements, the LD ratio should be low, which results in a low driving efficiency. Next, more detailed designs were analyzed in terms of performance. First, lightweight pods of the same weight were driven by magnetic levitation. Subsequently, pods of different weights were magnetically levitated at the same vertical displacement. The obtained performance results were compared.

First, when the lightweight pod mass was set to 1.35 ton, each design produced a required levitation force of 13.24 kN at different vertical displacements. The drag and stiffness corresponding to the vertical displacement are represented in Fig. 6 as blue, red, and black circles. In addition, these results are summarized in Table 3. Designs C1 and B1 generated higher lift forces than Design A1, thus confirming that pods can be levitated from a smaller vertical displacement. Therefore, the LD ratio was higher, which implies that driving based on magnetic levitation is more efficient than Design A1. Although Design A1 with similar vertical and horizontal stiffnesses can be relatively stable, Designs C1 and B1 have relatively low horizontal stiffnesses, which may be more affected by horizontal guideway irregularity.

**Table 3:** Comparison of the magnetic levitation/guidance characteristics of three designs for a pod with a mass of 1.35 tons moving at velocity ${v}_{h}$.

Design	$\Delta z$ (mm)	${F}_{z}$ (kN)	${F}_{x}$ (kN)	${k}_{y}$ (kN/m)	${k}_{z}$ (kN/m)	$\mathrm{LD}$ ratio
A1	– 47.1	13.24	– 0.1623	196.9	220.0	81.6
B1	– 35.9		– 0.0744	162.1	309.2	178.0
C1	– 33.9		– 0.0589	151.2	343.0	224.8

**Figure 7:**
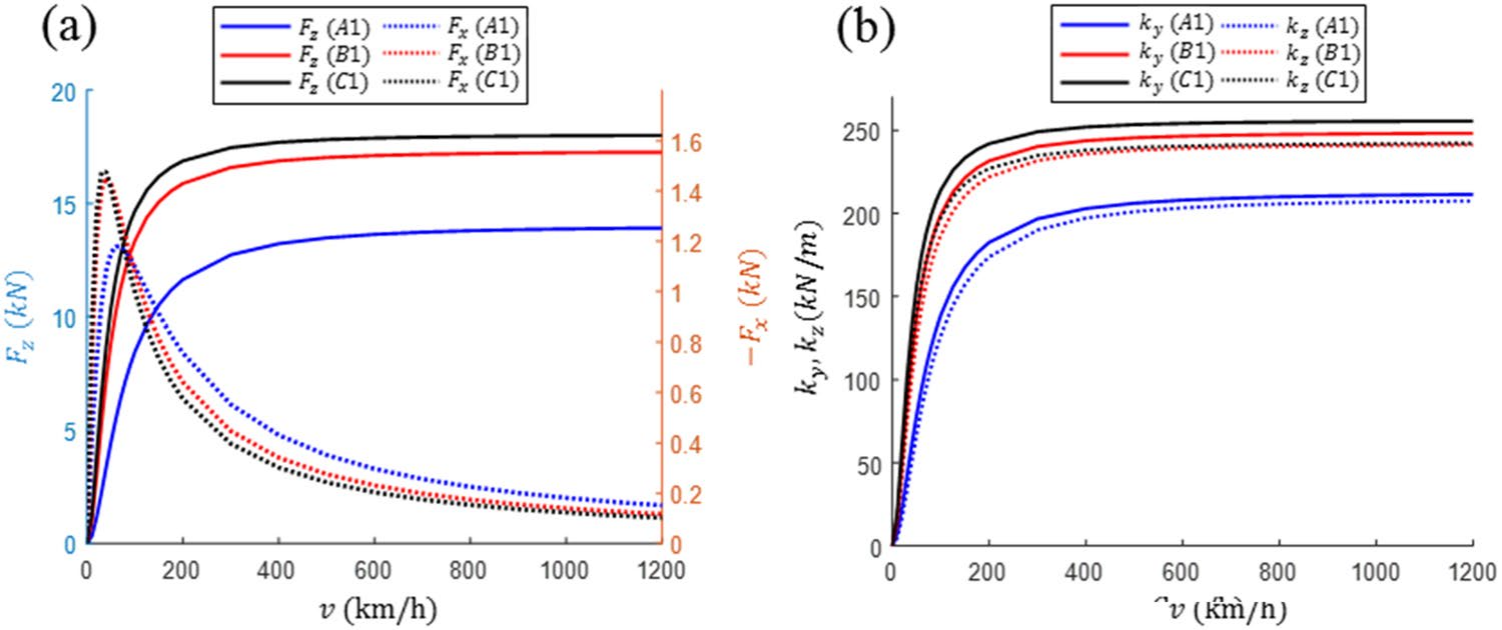
Comparison of the design characteristics with respect to changes in the driving velocity. (**a**) Lift and drag forces and (**b**) horizontal and vertical stiffnesses.

Thereafter, the characteristics of the three designs at the same vertical displacement were compared. For ∆z = – 50 mm, the lift and drag forces and the horizontal and vertical stabilities according to changes in the driving velocity were compared, as shown in Fig. 7 and Table 4. As can be observed, the lift force and the vertical/horizontal stiffness gradually increase with an increase in velocity, and then converge to a constant value. Moreover, the drag force results in an abrupt increase from a low rate to a maximum rate at 40–60 km/h, and then decreases rapidly with an increase in velocity. The performances of Designs B1 and C1 in terms of all the performance aspects of levitation/guidance are similar, with the ${F}_{z}$, ${k}_{y}$, and LD ratio of Design C1 being slightly superior. However, under the given SCMs and driving conditions, the performance of Design A1 is relatively inferior in all respects. The acceptable lifting weights of the A1, B1 and C1 designs are 1.41, 1.76, and 1.83 tons, respectively. The sizes or MMFs of the SCMs should be increased to allow for the boarding of passengers. For a comparison of the increase rate of the lift force at low velocities, the ratios of the lift forces at ${v}_{l}$ and ${v}_{h}$, denoted by ${F}_{zr}$, are listed in Table 4. As can be observed, the rates of ${F}_{zr}$ of Designs B1 and C1 are high and that of A1 is relatively low, which can be improved by reducing the resistance, e.g., by using thicker wires or lower resistivity materials.

**Table 4:** Comparison of the magnetic levitation/guidance characteristics of three designs when the magnetically levitated pods are moving at $\Delta z=$–50 mm for velocities ${v}_{h}$ and ${v}_{l}$.

Design	$\Delta z$ (mm)	$v$	${F}_{z}$ (kN)	${F}_{x}$ (kN)	${k}_{y}$ (kN/m)	${k}_{z}$ (kN/m)	$\mathrm{LD ratio}$	${F}_{zr}$(%)
A1	− 50	${v}_{h}$	13.87	0.1810	210.8	206.8	76.6	75.9
		${v}_{l}$	10.52	0.9104	167.5	157.3	11.6	
B1		${v}_{h}$	17.24	0.1394	247.9	240.8	123.7	87.4
		${v}_{l}$	15.07	0.8102	221.5	210.7	18.6	
C1		${v}_{h}$	17.99	0.1226	255.3	241.8	146.7	90.0
		${v}_{l}$	16.19	0.7345	233.4	217.8	22.0	

### Model validation with the FEM results

Next, we validate the EIM accuracy. First, the analysis results of different designs were compared with the FEM results to validate the applicability of the EIM to general EDS coil analysis. Thereafter, the analysis accuracies of the three selected designs were validated against the analysis results using FEM, followed by the accuracy of the EIM with respect to changes in ${P}_{e}$, which represents the number of adjacent coil pairs to be modeled. In addition, the accuracy of the induced EMF calculation with respect to changes in the number of loops was examined. For the validation analysis,${P}_{e}=3$ for the EIM and ${{n}_{turn}=N}_{turn}$ for the EMF computation were applied. Moreover, the errors of the analyzed results using the EIM and the FEM were evaluated by the following mean absolute percentage error (MAPE) for a total of *N* data points:

23 $$MAPE(\mathrm{\%})=\frac{1}{N}\sum_{i=1}^{N}\left|\frac{{f}_{i}-{\overline{f} }_{i}}{{\overline{f} }_{i}}\right|\times 100\mathrm{\%}$$

where ${f}_{i}$ and ${\overline{f} }_{i}$ are the data at the *i*-th data point computed by the EIM and FEM, respectively.

**Figure 8:**
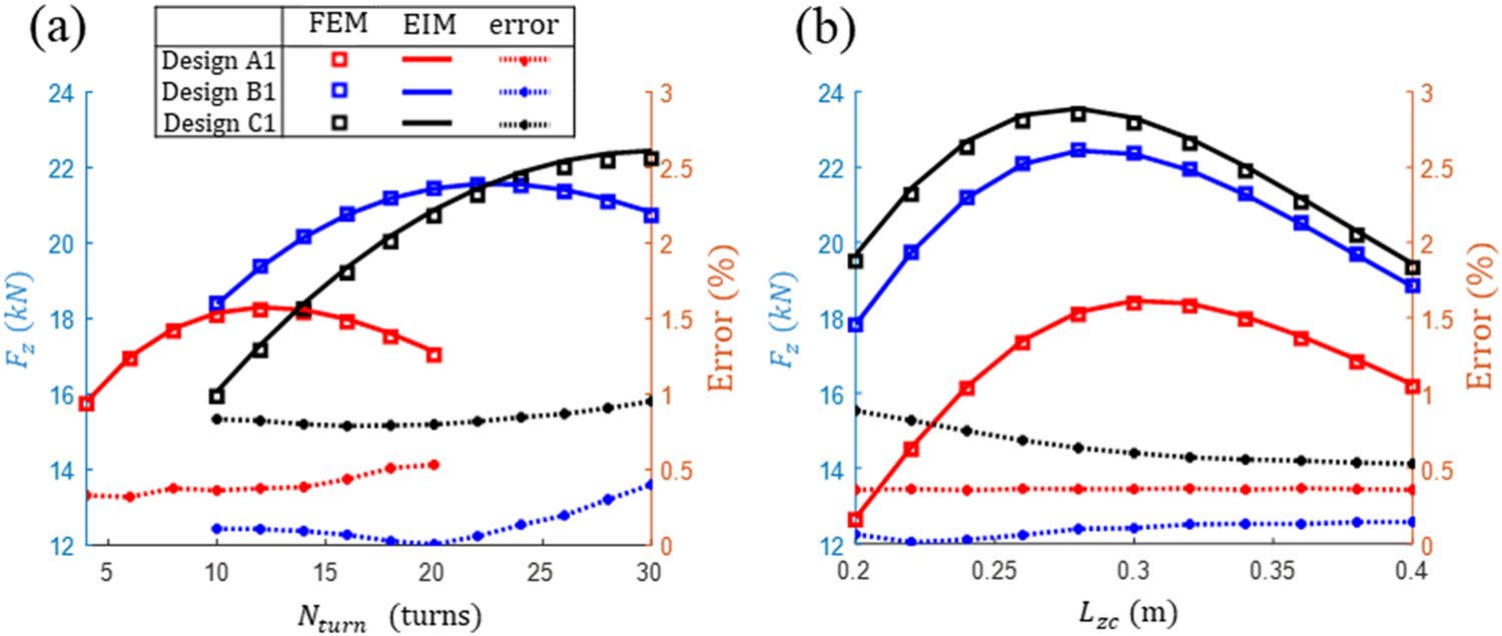
Comparison of the lift forces analyzed using the FEM and the EIM for different designs with respect to (**a**) the number of turns ${N}_{turn}$ and (**b**) the vertical coil height ${L}_{zc}$.

To validate the EIM, different ${N}_{turn}$ and ${L}_{zc}$ in Designs A1, B1, and C1 were considered. When driving at velocity ${v}_{h}$, the lift forces calculated at the maximum vertical displacement ${P}_{zm}$ for each design were compared with the FEM analysis results. The comparison results are shown in Fig. 8, where the errors evaluated for the FEM results are plotted on the right axis. As can be observed, ${F}_{z}$ evaluated for a total of 61 designs reveal that the values are consistent with respect to the design feature changes and that the errors in all cases are within 1%. Thus, it is confirmed that the EIM can be effectively utilized for various coil designs with varying sizes and ${N}_{turn}$ values, among other parameters.

**Figure 9:**
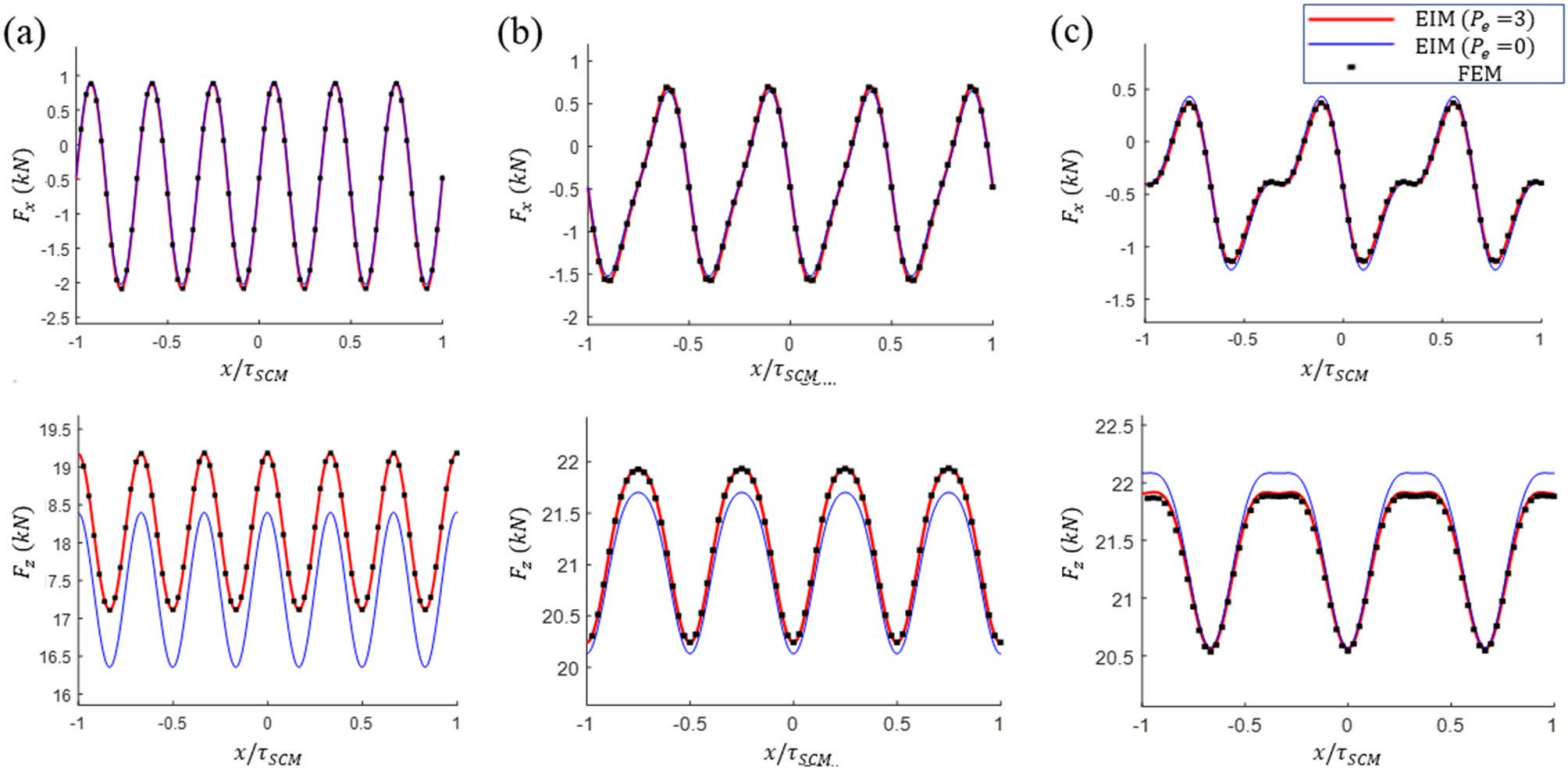
Comparison of the drag and lift force responses acting on the SCM driving in the ${P}_{zm}$ for the analysis results of Designs (**a**) A1, (**b**) B1, and (**c**) C1 based on the FEM and the EIM.

**Figure 10:**
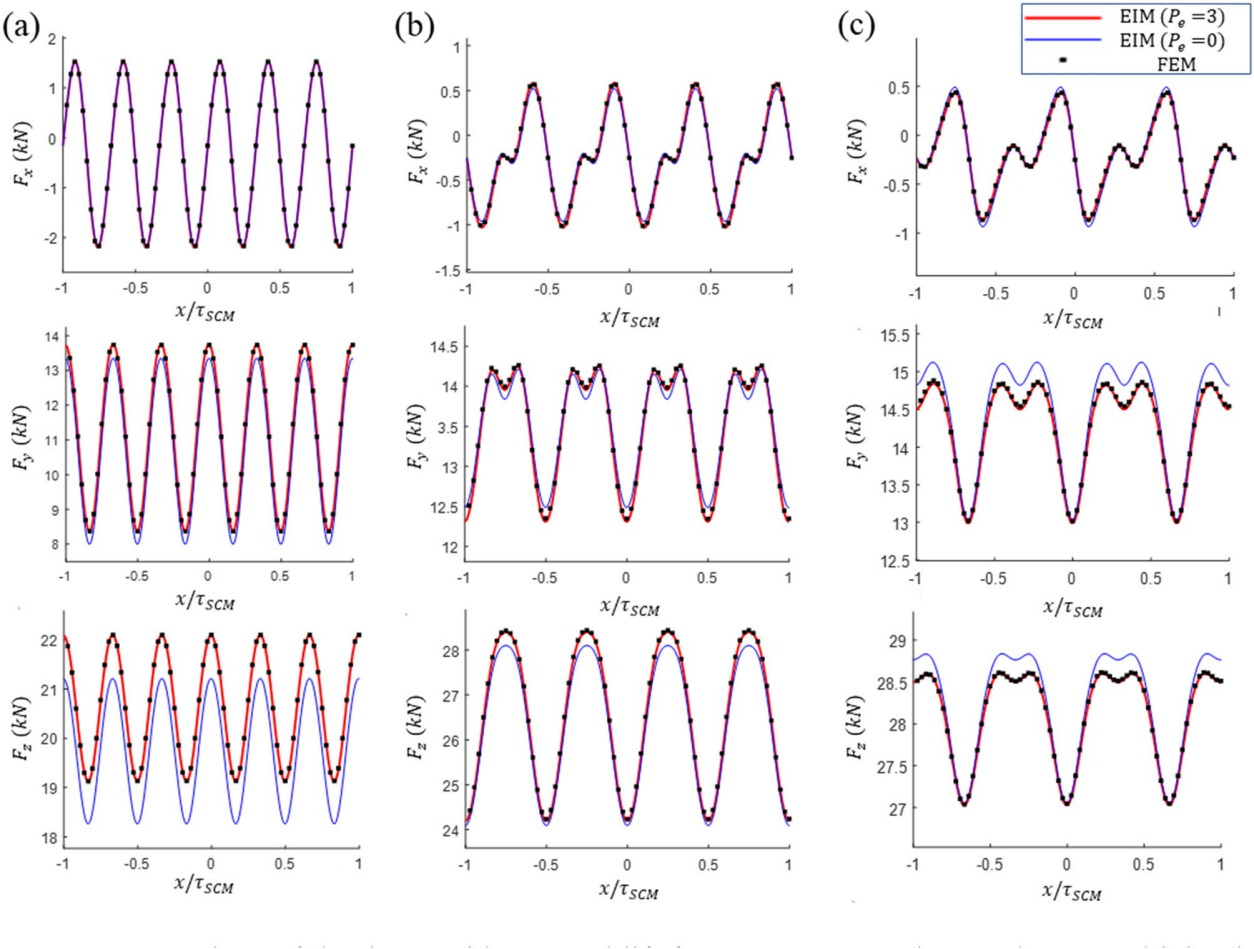
Comparison of the drag, guidance, and lift force responses acting on the SCM driving in ${P}_{zm}$ for the analysis results of Designs (**a**) A1, (**b**) B1, and (**c**) C1 based on the FEM and the EIM.

For a more detailed validation of the model, the analysis results for the drag, guidance, and lift force responses of the three designs were compared with the FEM results. Figures 9 and 10 present a comparison of the force responses at ${P}_{zm}$ and the maximum horizontal displacement ${P}_{ym}=({-\Delta \mathrm{y}}_{max}, -{\Delta \mathrm{z}}_{max}/2)$ with the FEM results. Furthermore, the calculated results for ${P}_{e}$ = 0 and ${P}_{e}$ = 3 are presented to compare the accuracy of the model according to the ${P}_{e}$ values. The analysis results for ${P}_{e}$ = 3 are consistent with the FEM analysis results in all cases. The analysis with ${P}_{e}$ = 0 yields a certain level of error; however, the waveform is similar. The computational errors of the EIM with respect to changes in the ${P}_{e}$ values are listed in Table 5. As can be observed, a relatively small drag results in a large number of near-zero data; therefore, the error calculations overestimate the actual difference. Although ${P}_{e}$ = 0 exhibits satisfactory analysis results and only includes the inductance effects in electrically connected null-flux coils, relatively large errors are observed in Designs A1 and C1. A high accuracy is achieved at ${P}_{e}$ = 1, which contains only the inductance effects of the closest pair of null-flux coils. In general, with an increase in ${P}_{e}$, the analysis error decreases. As ${P}_{e}$ is increased, the reaction forces converge to certain values, and the mutual inductance decreases abruptly as the coil distance is increased. Thus, it is computationally efficient to limit ${P}_{e}$ to a certain level, considering the level of convergence of the forces or the decrease in the mutual inductance.

**Table 5:** Comparison of the analysis accuracies for three models with respect to various ${P}_{e}$ values in the EIM.

Design	${P}_{e}$	Error (%) in ${P}_{zm}$		Error (%) in ${P}_{ym}$		
		${F}_{x}$	${F}_{z}$	${F}_{x}$	${F}_{y}$	${F}_{z}$
A1	0	14.665	4.258	4.937	3.541	4.259
	1	6.252	0.101	4.974	1.016	0.101
	2	6.191	0.078	4.990	1.065	0.130
	3	6.213	0.078	4.991	1.084	0.152
B1	0	14.079	0.795	16.328	0.671	0.888
	1	8.864	0.060	9.012	0.618	0.103
	2	8.872	0.034	9.077	0.591	0.157
	3	8.877	0.034	9.100	0.590	0.160
C1	0	38.319	0.592	20.604	1.238	0.496
	1	8.148	0.158	6.791	0.807	0.055
	2	7.452	0.140	7.020	0.794	0.049
	3	7.461	0.140	7.018	0.794	0.049

**Figure 11:**
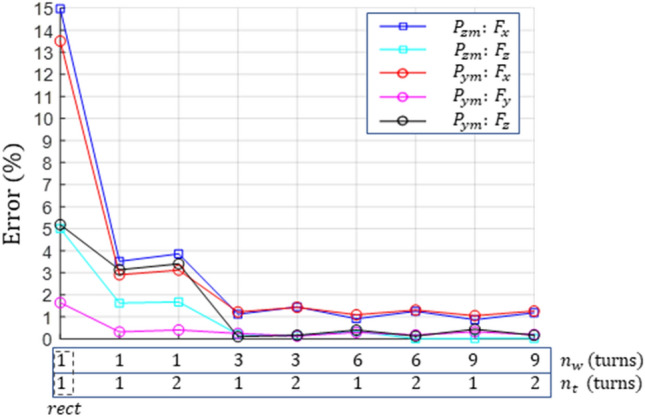
Comparison of the errors for the analyzed forces with respect to changes in the number of loops in Design B1.

The computational accuracies of the induced EMF with respect to changes in the number of closed-loops ${n}_{turn}={n}_{w}\times {n}_{t}$ were compared. For driving in ${P}_{zm}$ and ${P}_{ym}$ at ${v}_{h}$, the calculated force errors of Design B1 for the FEM results are shown in Fig. 11. The number of closed loops was changed from a single loop to ${n}_{w}=9, {n}_{t}=2,$ which was the same as the actual number of turns in Design B1. In addition, the simplest shape, namely, a rectangular single loop without a round corner, as denoted by *rect*, was considered. As mentioned previously, the ${F}_{x}$ errors were evaluated to be larger than the actual values. The lift force error for the rect loop was up to 5% and 3.4% for the single loop. For ${n}_{w}=3$ or a higher number of loops, the errors in the lift/guidance forces were sufficiently low (within 0.5%), and the forces for ${n}_{t}=2$ exhibited higher accuracies. Therefore, the coil shape in the EMF computation can be accurately explained, even at a lower number of turns ${n}_{turn}$ than the actual value ${N}_{turn}$.

## Experimental validation

**Figure 12:**
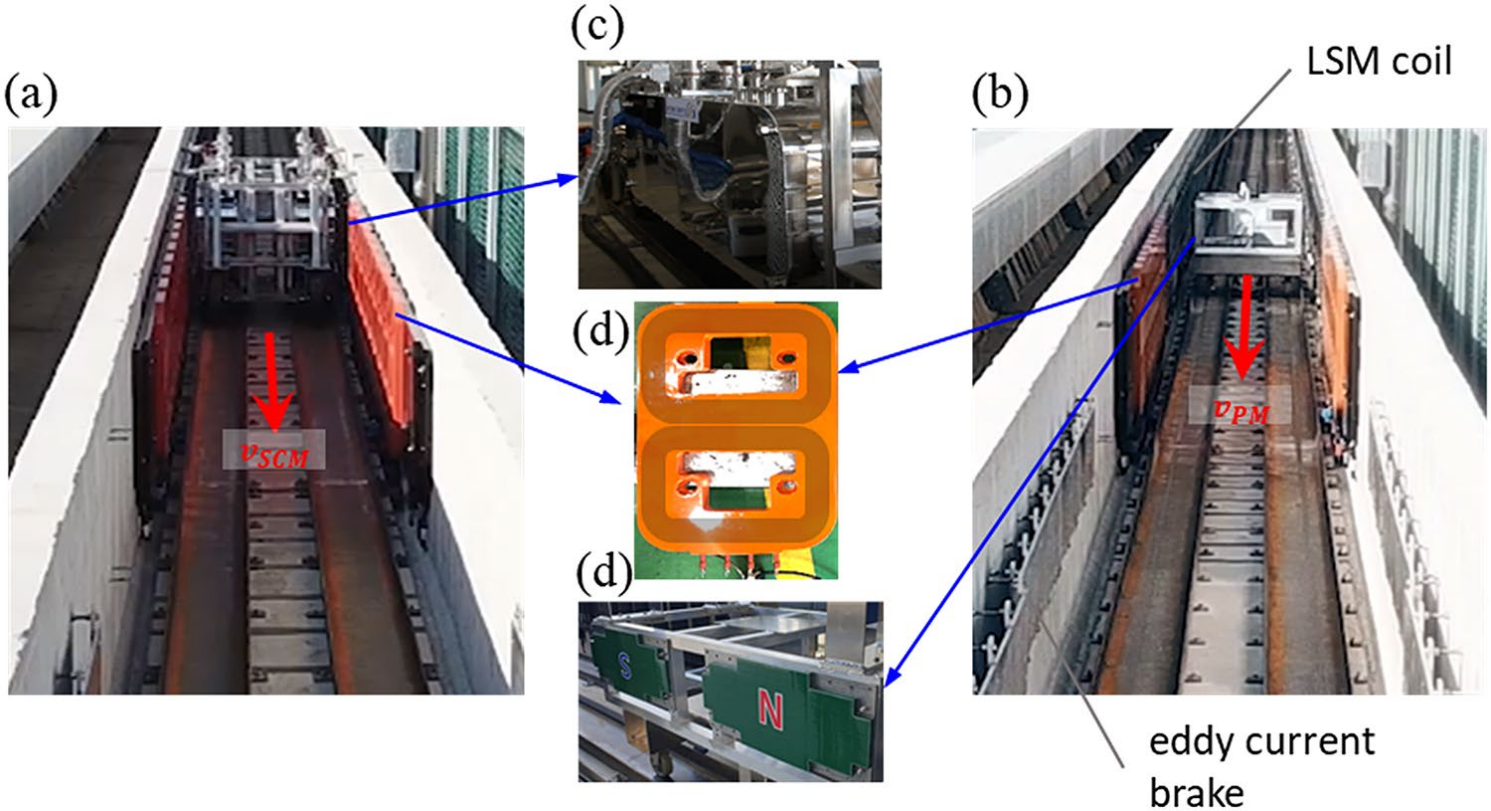
Induced current experiment of a null-flux coil in a small test bed for EIM validation: (**a**) SCM pod, (**b**) PM pod, (**c**) SCM module, (**d**) null-flux levitation coil, and (**d**) customized PM poles.

A small-scale testbed constructed for the LSM/EDS combination test of Hyperloop was utilized to validate the EIM model experimentally, as shown in Fig. 12. In the test bed, a pod was accelerated by an LSM installed in the section with a length of 60 m, and then braked by the eddy current induced on an aluminum plate in the rear section. In the experiment, two types of pods with the same pole pitch were used: one with 150 kAt of SCMs and another with N52 grade neodymium PMs. Moreover, the PM pole was customized to segment 78 block PMs to reduce the mass of the PM pod. In the testbed, 10 sets of the fabricated null-flux coils of the initial design were installed at a distance of 5.4 m near the maximum velocity section. The induced currents measured by four current sensors (LF 1005-S) attached to the sixth null-flux coil were used for validation. The tests were conducted at velocities ${v}_{SCM}$ and ${v}_{PM}$ for the SCM and PM pods, respectively. The detailed parameters related to the experiment are summarized in Table 6.

**Table 6:** Experimental parameters of PM bogie and null-flux coils in a small-scale testbed.

Parameter		Value	Unit
PM	The PM block size, $\mathrm{W}\times \mathrm{D}\times \mathrm{H}$	$0.04\times 0.04\times 0.02$	${\mathrm{m}}^{3}$
	The PM grade	$\mathrm{N}52$	–
	Number of PMs per pole	$78$	–
	Back iron thickness, ${d}_{t}$	$0.005$	$\mathrm{m}$
	Pole pitch, ${\tau }_{PM}$	$0.81$	m
	Experiment velocity, ${v}_{PM}$	$13.9 (50)$	m/s ($\mathrm{km}/\mathrm{h}$)
SCM	Coil size, ${L}_{\left(SCM\right)x}\times {L}_{\left(SCM\right)z}$	$0.6\times 0.3$	${\mathrm{m}}^{2}$
	Magnetomotive force, MMF	150	$\mathrm{kAt}$
	Pole pitch, ${\tau }_{SCM}$	$0.81$	m
	Experiment velocity, ${v}_{SCM}$	$13.1 (47)$	m/s ($\mathrm{km}/\mathrm{h}$)
Common	Number of poles, ${N}_{pole}$	4	–
	Pitch ratio, ${\tau }_{c}/{\tau }_{SCM}$	2/3	–
	Levitation coil ${L}_{xc}\times {L}_{zc}$	$0.426\times 0.3$	${\mathrm{m}}^{2}$
	Number of turns, ${N}_{turn}={N}_{w}\times {N}_{t}$	${N}_{w}=14, {N}_{t}=2$	–
	Air gap, ${g}_{air}$	$0.05$	$\mathrm{m}$
	Thickness of the levitation coil, ${L}_{t}$	$0.04$	m
	Wire cross sectional area, ${A}_{w}={c}_{w}\times {c}_{t}$	$6\times 15$	${\mathrm{mm}}^{2}$

**Figure 13:**
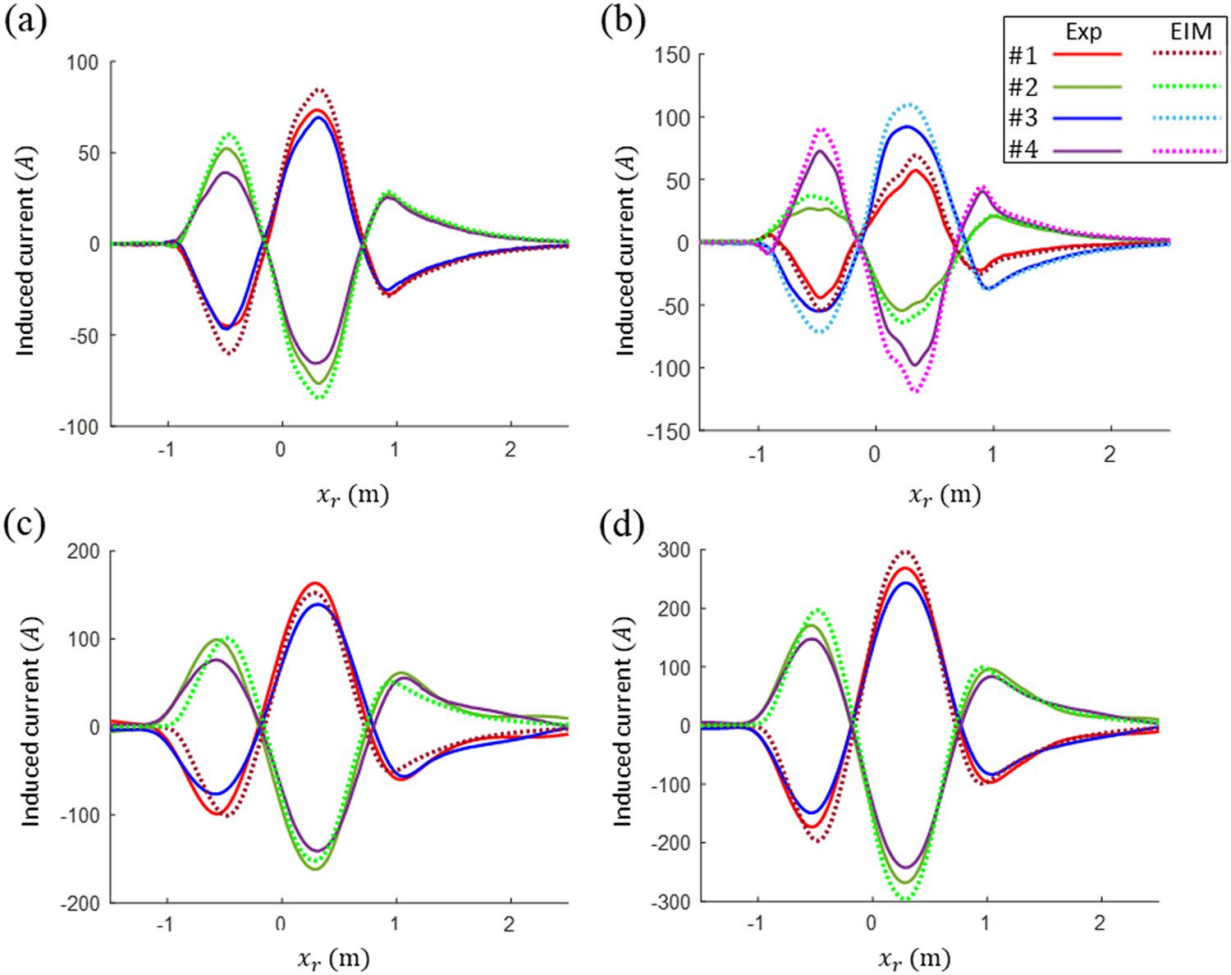
Comparison between the calculated induced current and the data measured in the experiment to validate the EIM: induced current in (**a**) ${P}_{z2}$ and (**b**) ${P}_{y}$ of the PM pod and induced current in (**c**) ${P}_{z1}$ and (**d**) ${P}_{z2}$ of the SCM pod.

The currents induced by the SCM pod were measured at ${P}_{z1}=\left(0, -30\mathrm{ mm}\right)$ and ${P}_{z2}=\left(0, -60\mathrm{ mm}\right)$, and the currents induced by the PM pod were measured at ${P}_{z2}$ and ${P}_{y}=\left(-30\mathrm{ mm}, -60\mathrm{ mm}\right)$. In addition, to reduce the measurement displacement error, all the induced current data were subtracted from the values measured at (0, 0). As shown in Fig. 13, the measured and analyzed currents at two different displacements for each pod were compared at the relative positions between the center positions of the pod and the measuring null-flux coil. In addition, *k* = 1–4 were assigned to the four coils of the null-flux coil from the top left to the bottom right.

Based on the results of the EIM validation, it was confirmed that the induced currents measured in the experiment and those calculated using the model were generally consistent with highly similar patterns. When the measurement results of ${P}_{z1}$ and ${P}_{z2}$ moving in the horizontal center were first verified, the induced currents in the coils at the same vertical position were expected to be the same as the EIM results. However, the measured results revealed a difference of approximately 10–15% for the maximum peak. In addition, given that the induced currents in the coils on the same side were similar, the actual vertical displacement on the left side of the pod was several millimeters lower than that on the right side. In addition, there was a difference of approximately 10–15% between the maximum peaks of the measured data and the maximum peaks of the analysis results. In the test of the PM pod at ${P}_{y}$, which exhibited a horizontal displacement, the measured currents of the four coils exhibited different patterns owing to the presence of guidance currents. The patterns at ${P}_{y}$ were similar to the analysis results, whereas the measured and analyzed data differed by approximately 18% for the maximum peak value.

There are several possible reasons for the difference in magnitude between the experimental data and the analysis results. The first is the driving position errors due to the horizontal and vertical guideway irregularities. In addition, the actual induced current might have been low because of the contact resistance at the null-flux connection terminal. Next, for the PM pod, the velocity of the measured induced current waveform was in good agreement with the analysis result. In contrast, the SCM pod exhibited a small difference of approximately 30 mm for the zero intersection, which can be attributed to the pole position errors of the SCMs. The maximum induced current and the averaged lift force analyzed at ${P}_{z2}$ with the same displacement of the two pods were compared. From the comparison, the SCM pod with an MMF of 150 kAt yielded 296.3 A and 3.0 kN, respectively, and the PM pod yielded 84.7 A and 0.24 kN, respectively. Thus, the MMF of the PM pod was estimated as 43 kAt. However, considering that the measured induced currents were slightly lower in the actual experiment, the actual lift and guidance forces were slightly lower than expected.

## Conclusions

In this paper, an EIM is presented for the rapid and accurate analysis of an EDS system. The EIM treats the magnetic coupling effects of adjacent coils as an equivalent inductance and provides the user with decoupled RL equations for isolated coils. The EIM for normal- and null-flux coils is described by utilizing the relationship of the induced current and the EMF between levitation coils arranged at regular intervals. We used the proposed EIM to design null-flux coils for SC-EDS in Hyperloop. As a result, three designs with satisfactory levitation forces and improved horizontal stiffnesses were obtained. The characteristics of the obtained designs were compared. In addition, the analysis accuracy of the EIM with respect to changes in ${P}_{e}$ and the number of loops was validated. The EIM exhibited a considerably high error accuracy within 1% when compared with the FEM analysis results for various designs. For two types of moving pods: SCMs and PMs on a small-scale testbed, the measured current waveforms on the levitation coils were in good agreement with the analysis results. However, several differences in the wave peak could be attributed to the position error of the pod or the contact resistances of the coils. A rapid and accurate analysis using the proposed EIM can be effectively utilized for the general analysis and design of coil-based EDS levitation systems and multiphysics simulations such as heat transfer analysis of coils and vibration analysis during magnetic levitation.

## References

[1] Hyperloop Alpha. https://www.tesla.com/sites/default/files/blog_images/hyperloop-alpha.pdf. Accessed 1 Sept 2021.

[2] Hamad, A., Izabela, K. & John P. Hyperloop-prediction of social and physiological costs. *Trans. Syst. Tech.* (2020).

[3] Gkoumas K, Christou M (2020). A triple-helix approach for the assessment of hyperloop potential in Europe. Sustainability.

[4] Gkoumas K (2021). Hyperloop academic research: A systematic review and a taxonomy of issues. Appl. Sci..

[5] Nøland JK (2021). Prospects and challenges of the hyperloop transportation system: A systematic technology review. IEEE Access.

[6] Mitropoulos L (2021). The hyperloop system and stakeholders: A review and future directions. Sustainability.

[7] Shanghai Maglev Transportation Development Co., Ltd Website. http://www.smtdc.com/en. Accessed 1 Sept 2021.

[8] Central Japan Railway Company Website. https://scmaglev.jr-central-global.com. Accessed 1 Sept 2021.

[9] Sotelo GG (2014). A full scale superconducting magnetic levitation (MagLev) vehicle operational line. IEEE Trans. Appl. Supercond..

[10] Deng Z (2017). A high-temperature superconducting maglev-evacuated tube transport (HTS Maglev-ETT) test system. IEEE Trans. Appl. Supercond..

[11] Hardt Website. https://hardt.global/technology-development/. Accessed 1 Sept 2021.

[12] Zeleros Website. https://zeleros.com/. Accessed 1 Sept 2021.

[13] Transpod Website. https://www.transpod.com/technology-demonstrator/. Accessed 1 Sept 2021.

[14] Nevono Website. Available online: https://www.nevomo.tech/en/. Accessed 1 Sept 2021.

[15] Hyperloop Transportation Technologies Website. https://www.hyperlooptt.com/technology/. Accessed 1 Sept 2021.

[16] Virgin Hyperloop website. https://virginhyperloop.com/.

[17] Choi SY (2019). Sub-sonic linear synchronous motors using superconducting magnets for the hyperloop. Energies.

[18] Lee J (2021). Development of the reduced-scale vehicle model for the dynamic characteristic analysis of the hyperloop. Energies.

[19] Lim J (2020). Design optimization of a 2G HTS magnet for subsonic transportation. IEEE Trans. Appl. Supercond..

[20] Mun J (2021). Thermal and electromagnetic performance evaluation of REBCO magnet with solid nitrogen thermal battery for maglev train. IEEE Trans. Appl. Supercond..

[21] Powell J. R., Jr. & Danby, G. T. Electromagnetic inductive suspension and stabilization system for a ground vehicle. U.S. Patent No. 3,470,828. (1969).

[22] Hieronymus H, Miericke J, Pawlitschek F, Rudel M (1974). Experimental study of magnetic forces on normal and null flux coil arrangements in the inductive levitation system. Appl. Phys..

[23] Miericke J, Urankar L (1973). Theory of electrodynamic levitation with a continuous sheet track: Part I. Appl. Phys..

[24] Lever, J. H. Technical assessment of maglev system concepts. Final report. No. AD-A-358293/XAB.; CRREL-SR-98-12. Cold Regions Research and Engineering Lab, 1998. https://rosap.ntl.bts.gov/view/dot/42287/dot_42287_DS1.pdf. Accessed 3 Aug 2020.

[25] Lim S, Kim D, Yoo J, Park NC (2019). A method of induced current estimation of EDS type maglev using 3D transient EM analysis. Trans. Korean Soc. Mech. Eng..

[26] Guo Z, Li J, Zhou D (2019). Study of a null-flux coil electrodynamic suspension structure for evacuated tube transportation. Symmetry.

[27] Gong T (2021). 3-D FEM modeling of the superconducting EDS train with cross-connected figure-eight-shaped suspension coils. IEEE Trans. Appl. Supercond..

[28] Burkhardt EE, Schwartz J, Nakamae S (1993). Analysis of superconducting magnet (SCM)-ground coil interactions for EDS Maglev coil configurations. IEEE Trans. Appl. Supercond..

[29] He JL, Rote DM, Coffey HT (1993). Applications of the dynamic circuit theory to maglev suspension systems. IEEE Trans. Magn..

[30] Davey KR (1997). Designing with null flux coils. IEEE Trans. Magn..

[31] He JL, Coffey HT, Rote DM (1995). Analysis of the combined maglev levitation, propulsion, and guidance system. IEEE Trans. Magn..

[32] Cai Y (2020). Semianalytical calculation of superconducting electrodynamic suspension train using figure-eight-shaped ground coil. IEEE Trans. Appl. Supercond..

[33] Semail E, Bouscayrol A, Hautier J-P (2003). Vectorial formalism for analysis and design of polyphase synchronous machines. EPJ Appl. Phys..

[34] Sellam A (2013). A vectorial modeling for the permanent magnet synchronous machine (polyphase) based on multimachine approach. Int. J. Electr. Eng. Inform..

[35] Lim J (2020). Design model of null-flux coil electrodynamic suspension for the hyperloop. Energies.

[36] Simcenter MAGNET website. https://www.plm.automation.siemens.com/global/en/products/simcenter/magnet.html. Accessed 1 Sept 2021.

[37] Yoon R (2021). Capsule vehicle dynamics based on levitation coil design using equivalent model of a sidewall electrodynamic suspension system. Energies.

